# Changes in oligodendroglial subpopulations in Parkinson’s disease

**DOI:** 10.1186/s13041-023-01055-5

**Published:** 2023-09-14

**Authors:** Eun-Jin Bae, Dayana Pérez-Acuña, Ka Hyun Rhee, Seung-Jae Lee

**Affiliations:** 1 Department of Biomedical Sciences, Seoul National University College of Medicine, 103 Daehak-ro, Jongro-gu, Seoul, 03080 South Korea; 2 Neuroscience Research Institute, Seoul National University College of Medicine, Seoul, South Korea; 3 Convergence Research Center for Dementia, Seoul National University College of Medicine, Seoul, South Korea; 4 Neuramedy Co., Ltd, Seoul, South Korea; 5 Present Address: Department of Biochemistry, Molecular Biology and Biophysics, University of Minnesota, Minneapolis, MN 55455 USA

**Keywords:** Parkinson’s disease, Oligodendrocytes, Myelination, Single nuclei transcriptomics

## Abstract

Parkinson’s disease (PD) is characterized by a selective loss of dopaminergic neurons. While most research on PD conducted to date has focused on neurons and, to a certain extent, glia, few studies have investigated changes in oligodendroglia. Here, we investigated the heterogeneity of oligodendrocytes from PD patients compared with those of control cases by analyzing single-nuclei transcriptomes. These analyses revealed the presence of distinct oligodendrocyte populations in PD patients indicative of corresponding variations in molecular features, notably including activation of inflammatory responses, response to protein folding stress, and myelination abnormalities. We confirmed myelination abnormalities in an α-synuclein preformed fibril-injection mouse model of PD. These results suggest that oligodendrocytes acquire disease-associated phenotypes in PD and may contribute to the accompanying neurodegeneration.

**Supplementary Information:**

The online version contains supplementary material available at 10.1186/s13041-023-01055-5.

Parkinson’s disease (PD) is a progressive neurogenerative disease characterized by movement defects, including resting tremor, bradykinesia, and rigidity. Selective loss of dopaminergic neurons in the substantia nigra pars compacta and the formation of inclusion bodies known as Lewy bodies are the characteristic pathological features of PD [1]. Given that dopaminergic neurons in the midbrain are involved in the regulation and coordination of movement, selective loss of these cell populations has been regarded as a cause of the motor control abnormalities observed in PD patients [2].

A growing body of evidence has revealed that glial cells are intimately involved in brain disorders, including Alzheimer’s disease and PD. Hyperactivation of astrocytes and microglia leads to chronic neuroinflammation, an important contributor to neurodegeneration [3, 4]. Notably, single-cell transcriptomics of the substantia nigra and the cortex from five human postmortem brains showed enrichment of PD-associated genes not only in nigral dopaminergic neurons but also in oligodendrocytes [5]. In addition, a single-nuclei transcriptomic analysis of post-mortem midbrains from PD patients showed a decrease in the number of oligodendrocytes positive for OPALIN, a marker for differentiating oligodendrocytes [6]. Although these previous results clearly imply disease-associated changes in oligodendrocytes in PD, the phenotypic changes that oligodendrocytes undergo during the PD pathogenesis have not been properly analyzed.

In this study, we analyzed single-nuclei transcriptomes of the putamen from four control cases and four PD patients previously published by Xu et al. [7] to unveil cellular diversities of oligodendrocytes linked with PD. To classify the various types of cells in the putamen, we pre-clustered all cells from the eight samples, yielding a dataset of 19,076 nuclei. Using the UMAP algorithm, we embedded all nuclei—7767 from control individuals and 11,309 from PD patients—into two dimensions. Employing the Panglao database and a two-tailed Fisher’s exact test, we identified and annotated the following major cell types in the human putamen: oligodendrocytes (marked by MBP), astrocytes (AQP4), excitatory neurons (NRGN), oligodendrocyte progenitor cells (OLIG1), and microglia (CSF1R). Including subtypes, human putamen tissues are composed of 10 major cell types (96.27% of all cells) (Fig. 1A). To investigate transcriptomic differences between control and PD oligodendrocytes, we extracted oligodendrocyte fractions and re-clustered these nuclei, identifying five major subpopulations in the oligodendrocyte fraction (Fig. 1B, Additional file 1: Table S1). We found that cluster 3 was barely detectable in PD samples, but was the major subpopulation in control cases, accounting for 38.97% of oligodendrocytes (Fig. 1C). In contrast, PD samples showed about a threefold increase in cell number in clusters 1 and 2 (Fig. 1C). Given that the percentage of cluster 0 in PD samples was comparable to that in control cases, we postulated that oligodendrocytes in cluster 3 transform into either cluster 1 or 2 oligodendrocytes during the pathogenesis of PD.

**Fig. 1:**
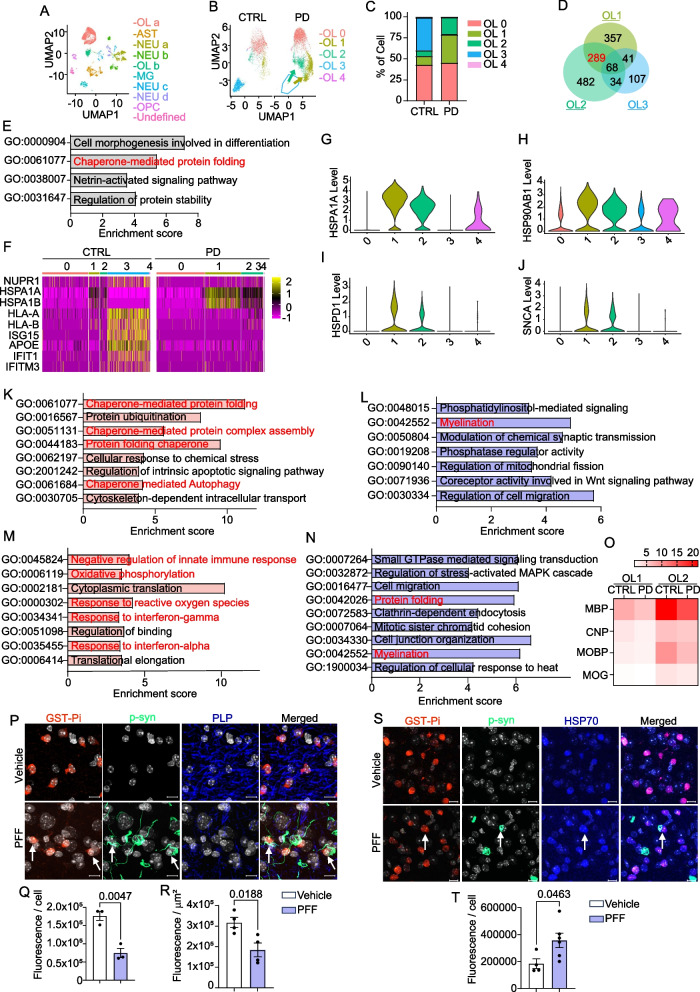
Single-nuclei RNA-seq identifies subsets of oligodendrocytes in PD. **A** UMAP depicting cell subsets in the striatum from control cases and PD patients. **B** UMAP depicting oligodendroglial subpopulations from control cases and PD patients. Each dot represents a cell. Cells are color coded based on their cluster affiliation. **C** Composition of oligodendrocyte subpopulations in control and PD. **D** Venn diagrams displaying the number of featured genes in clusters 1–3. **E** Enriched GO terms for common featured genes of clusters 1 and 2. **F** Heatmap showing differences in clusters based on the most variable genes (FDR < 0.05) in control and PD samples. Scale: Z-score. **G**–**J** Violin plots showing expression of marker genes associated with molecular chaperoning. **K** Enriched GO terms among DEGs increased in cluster 1. **L** Enriched GO terms among DEGs decreased in cluster 1. **M** Enriched GO terms among DEGs increased in cluster 2. **N** Enriched GO terms among DEGs decreased in cluster 2. **O** Heatmap showing average expression of myelinating genes in clusters 1 and 2 in control and PD samples. Scale: average of each gene expression. **P** Immunofluorescence staining of phosphorylated synuclein (p-syn; green), PLP (blue), and GST-Pi (red) in the putamen in vehicle or α-synuclein PFF-injected mice. Scale bar: 10 µm. **Q**, **R** Quantification of mean fluorescence intensity of GST-Pi (Q) and PLP (R). Each point represents a single mouse. Data are expressed as means ± SEM. **S** Immunofluorescence staining of phosphorylated synuclein (p-syn; green), HSP70 (blue), and GST-Pi (red) in the putamen in vehicle or α-synuclein PFF-injected mice. Scale bar: 10 µm. **T** Quantification of mean fluorescence intensity of HSP70 in GST-Pi positive cells

To further characterize PD-associated oligodendrocytes (clusters 1 and 2), we analyzed cluster-enriched sets of feature genes in clusters 1–3. We discovered 289 genes that were shared by clusters 1 and 2, but not by cluster 3. These genes are linked to the following Gene Ontology (GO) terms: chaperone-mediated protein folding, netrin-activated signaling pathway, regulation of protein stability, and cell morphogenesis involved in differentiation, suggesting that oligodendrocytes are affected by protein folding stress in PD (Fig. 1D and E, and Additional file 2: Table S2). Consistent with this, there was a detectable increase in the expression of molecular chaperones in clusters 1 and 2 in PD samples, implying that clusters 1 and 2 undergo the protein folding stress (Fig. 1F–J).

Next, to identify the molecular features of oligodendroglial subpopulations linked to PD, we analyzed enriched pathways among differentially expressed genes (DEGs) in clusters 1 and 2. We found that chaperone-mediated protein folding and autophagy were enriched in cluster 1, the largest oligodendroglial cluster in PD samples (Fig. 1K, Additional file 2: Table S2). This finding is in line with the hypothesis that oligodendrocytes are influenced by protein folding stress in PD. In contrast, cluster 2 showed an increase in interferon response, oxidative phosphorylation, and negative modulation of the innate immune response (Fig. 1M, Additional file 2: Table S2), suggesting that this oligodendroglial subpopulation undergoes inflammatory reprogramming in PD patients. We also found that myelination was reduced in both of these clusters (Fig. 1L and N, and Additional file 2: Table S2). Notably, PD samples showed a marked decrease in the expression of myelination-related genes in clusters 1 and 2 (Fig. 1O). These results collectively suggest that oligodendrocytes in PD undergo protein folding stress and inflammatory responses, and exhibit reduced myelination.

To further validate these findings, we assessed oligodendroglial synucleinopathies and myelin abnormalities in an α-synuclein preformed fibril (PFF)-injection mouse model of PD. We found pathological α-synuclein aggregates in oligodendrocytes in the striatum at 20 weeks post-injection (Fig. 1P), as well as a reduction in myelinating protein PLP (Fig. 1P, R and Additional file 3: Fig. S1). Glutathione-S-transferase Pi form (GST-Pi), a marker for mature oligodendrocytes, was also decreased in this synucleinopathy model (Fig. 1P, Q and Additional file 3: Fig. S1). These findings suggest that abnormal deposition of pathological α-synuclein aggregates may cause defects in oligodendrocyte maturation and myelination. Mice injected with α-synuclein PFF also showed the elevation of HSP70 in GST-Pi positive oligodendrocytes, indicating an increased protein folding stress in oligodendrocytes (Fig. 1S, T).

Here, we identified oligodendrocyte subpopulations linked to PD by analyzing human single-nuclei transcriptome data from the striatum of control cases and PD patients and validated the presence of these oligodendroglial subpopulations in a mouse model. Our enrichment studies revealed that oligodendrocyte subpopulations linked to PD exhibit features of protein folding stress and inflammatory reprogramming, as well as a decrease in myelination-related genes. These features were further confirmed in the α-synuclein PFF mouse model. These findings highlight the molecular pathways that are related to the pathogenic changes in oligodendrocytes that occur during the pathogenesis of PD. The PFF mouse model exhibits α-synuclein aggregates in oligodendrocytes over time [8]. The oligodendroglial synucleinopathy indeed occur in human PD [9], justifying the use of the PFF model for validation. However, this oligodendroglial pathology is not a prominent feature of human PD. Therefore, our interpretation of the current results will need additional validation in human brain tissues. Despite this limitation, our independent analysis of this dataset is consistent with a prior study showing a decrease in OPALIN-high cells in post-mortem PD brain [6]. Our findings suggest that oligodendrocytes, like other brain cell types, acquire disease-specific molecular signatures in PD, including inflammation reprograming and myelination abnormalities, and provide the basis for future research into how this PD-associated oligodendroglial diversity contributes to PD.

### Supplementary Information

**Additional file 1: Table S1.** UMAP coordinates of each cell.

**Additional file 2: Table S2.** Adjusted p-values and associated gene lists of enriched GO terms in Fig. 1 E, K, L, M, and N.

**Additional file 3. Figure S1** and materials and methods.

## Data Availability

The metadata for Xu et al. [7] (accession number GSE161045) were downloaded from Gene Expression Omnibus (GEO). All materials are available from the corresponding author upon reasonable request.

## Abbreviations

GO: Gene Ontology
PD: Parkinson’s disease
PFF: Preformed fibril
