# Supplementary material for: Changes in oligodendroglial subpopulations in Parkinson’s disease
Source: Mol Brain. 2023 Sep 14;16:65. doi: 10.1186/s13041-023-01055-5 (PMC10500805; doi:10.1186/s13041-023-01055-5)
Supplement: Supplementary file 3 — Additional file 3. Figure S1 and materials and methods. [file 13041_2023_1055_MOESM3_ESM.docx]

**Additional file**

**Changes in oligodendroglial subpopulations in Parkinson’s disease**

Eun-Jin Bae ^1,2*^, Dayana Pérez-Acuña ^1^, Ka Hyun Rhee ^1^, and Seung-Jae Lee^1,2,3,4*^

^1^ Department of Biomedical Sciences, Seoul National University College of Medicine, Seoul 03080, Korea

^2^ Neuroscience Research Institute, Seoul National University College of Medicine, Seoul, South Korea

^3^ Convergence Research Center for Dementia, Seoul National University College of Medicine, Seoul, South Korea

^4^ Neuramedy Co., Ltd, Seoul, South Korea

* Corresponding authors: Seung-Jae Lee ([sjlee66@snu.ac.kr](mailto:sjlee66@snu.ac.kr)) or Eun-Jin Bae ([agarci@hanmail.net](mailto:agarci@hanmail.net)); Department of Biomedical Sciences, Seoul National University College of Medicine, 103 Daehak-ro, Jongro-gu, Seoul 03080, Korea; Tel: +82-2-3668-7037; Fax: +82-2-447-5683

Present Address: Ka Hyun Rhee, Department of Biochemistry, Molecular Biology and Biophysics, University of Minnesota, Minneapolis, MN, 55455, USA

**Additional Figure**

**
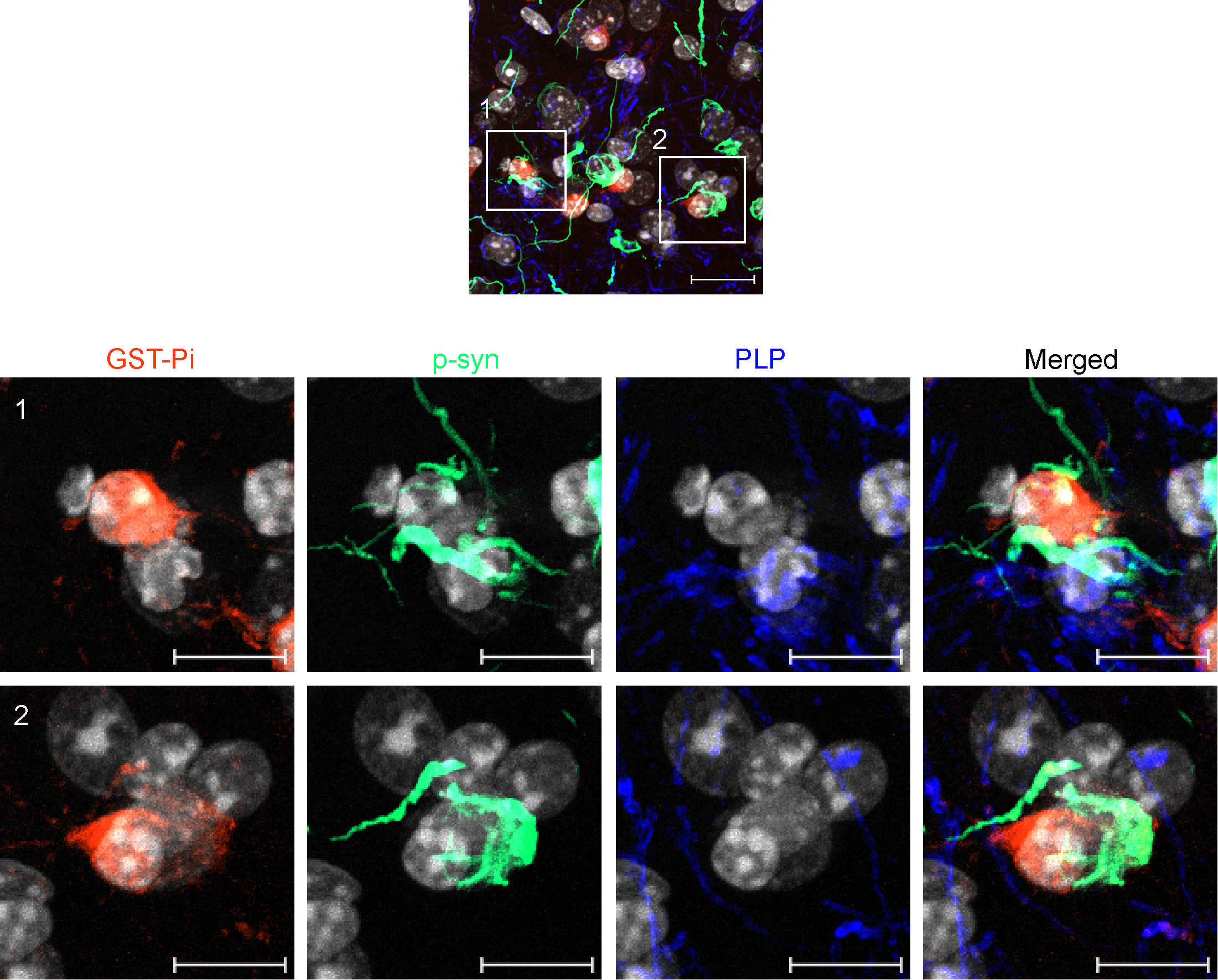
**

**Figure S1. Magnified images of lower panels in Figure 1P.**

Magnified immunofluorescence images of phosphorylated synuclein (p-syn), PLP, and GST-Pi in the putamen in α-synuclein PFF-injected mice. Red, GST-Pi; green, phosphorylated α-synuclein; blue, PLP; white, DAPI. Scale bar: 10 μm.

**Materials and Methods**

**Mapping snRNA-seq data to the reference genome and quality control**

The dataset was obtained from Xu et al [1]. R version 4.2.2 was used for statistical analyses and plotting. Filtered gene-cell barcoded matrices, generated with CellRanger 3.0.2 (10x Genomics) based on the human GRCh38 pre-mRNA reference genome, were used for further analysis with the R package, Seurat v4.3.0 [2]. For quality control, nuclei with less than 200 genes, more than 5% mitochondrial content or an extremely high number of detected genes or UMIs (unique molecular identifiers) were filtered out. After application of the quality control process, 19,076 single-nucleus expression profiles were obtained.

**Cell clustering and subcluster analysis**

The data integration process was performed according to Seurat instructions. The function, SelectIntegrationFeatures (nfeatures = 3,000), was used to identify variable genes. The top 3,000 variable genes were used for integration-features and integration-anchor selection. Clusters were identified with the functions, FindNeighbors (dims = 1:10) and FindClusters (resolution = 0.15). Marker genes were identified using the function, FindAllMarkers (min.pct = 0.25, og2fc.threshold = 0.3). A P-value < 0.05 was used as a cutoff for determining marker genes. Cell types—oligodendrocytes (marked by MBP, MOG, PLP1), astrocytes (AQP4, GFAP), excitatory neurons (NRGN SYT1, GRIA1), oligodendrocyte progenitor cells (OLIG1, OLIG2), and microglia (CSF1R, AIF1)—were annotated based on the Panglao database [3]. The function, subset, was used to isolated nuclei of oligodendrocyte clusters (control = 7,767; PD = 11309 nuclei) from the original Seurat object. After data integration and unsupervised clustering with a resolution of 0.2, marker genes were identified using the function, FindAllMarkers (min.pct = 0.25, og2fc.threshold = 0.3). Significantly differentially expressed genes (DEGs) were defined as those with a Benjamini–Hochberg (FDR) adjusted *P*-value < 0.05 and a log2 of fold change > 0.3 or < −0.3. Enrichment analyses of Gene Ontology terms were performed using the Cytoscape plug-in, ClueGO, based on related terms and statistical significance [4].

**Generation of recombinant α-synuclein and preparation of mouse PFFs**

For purification of mouse wild-type (WT) α-synuclein, *Escherichia coli* strain BL21 (DE3) was transformed with a pDdulGC vector encoding mouse WT α-synuclein and grown in LB medium at 37°C. After cells reached an optical density at 600 nm (OD_600_) of 0.6–0.7, protein expression was induced by incubation with 0.1 mM isopropyl β-D-1-thiogalactopyranoside for 3 hours at 37°C. Cells were harvested by centrifugation at 4000 × g for 15 minutes at 4°C and sonicated. Following centrifugation of lysates at 13,999 rpm (Supra R22, A50-8 rotor; Hanil Scientific (Gimpo, South Korea) for 20 minutes at 4°C, the supernatant was boiled for 10 minutes, then centrifuged again under the same conditions. The resulting supernatant was filtered through 0.22-μm membranes and purified, first by anion exchange chromatography (HiTrap Q FF column; GE Healthcare Life Sciences, Chicago, IL, USA) and then by size-exclusion chromatography of eluted fractions containing α-synuclein (Hiload 16/600 Superdex 200 pg column; GE Healthcare Life Sciences, Chicago, IL, USA). Purified fractions were pooled, dialyzed against deionized water, and lyophilized.

Lyophilized α-synuclein was reconstituted in Dulbecco’s phosphate-buffered saline (a1285601; Carlsbad, CA, USA). Aggregates were removed by filtering reconstituted α-synuclein through a 100-kDa membrane (Nanosep, OD100C34; Pall Life Sciences, Port Washington, NY, USA), then incubated at 37°C for 7 days with constant shaking at 1,000 rpm. PFFs were stored at ‑80°C until the day of injection, at which time the fibrils were thawed at room temperature and sonicated for a total of 30 seconds (60 pulses, 1 second on, 1 second off) at 20% power (Vibracell VCX130; Sonics, Newtown, CT, USA).

**Animals and stereotaxic injection of PFFs**

All animal experiments were performed on 8-week-old, male WT C57BL/6N mice in accordance with the standards of the Seoul National University Institutional Animal Care and Use Committee (IACUC; SNU-190417-2-6). Mice were anesthetized by intraperitoneal injection of ketamine/xylazine. A total volume of 2.5 µl of phosphate-buffered saline (PBS) or PFFs (2 mg/ml; 5 µg total) was stereotaxically injected into the right striatum (AP, 1.0 mm; ML, 1.5 mm; DV, 3.0 mm) at a rate of 0.5 µl/min using a 30G needle.

**Sample collection**

Twenty weeks after intrastriatal injection, mice were anesthetized with ketamine hydrochloride and xylazine hydrochloride (3.5:1, 2.5 µl/g) and then transcardially perfused with saline, followed by ice-cold 4% paraformaldehyde (PFA). Brains were dissected out and fixed in PBS containing 4% PFA for at least 48 h at 4°C for neuropathological analysis.

**Immunofluorescence staining**

Epitope retrieval was carried out by heating sections at 95°C in sodium citrate buffer (10 mM plus 0.05% Tween-20, pH 6.0) for 5 minutes. Free-floating brain sections were blocked by incubating with PBS containing 0.05% Triton X-100 (PBST), 4% bovine serum albumen (BSA) and 5% normal goat serum, followed by incubation with primary antibodies overnight at 4°C. Anti-phosphorylated α-synuclein mouse monoclonal antibody (P-syn/81A; Abcam, Cambridge, MA, USA), anti-phosphorylated α-synuclein rabbit monoclonal antibody (EP1536Y; Abcam), anti-myelin PLP rabbit polyclonal antibody (ab28486; Abcam), anti-human GSTP1 goat polyclonal antibody (LS-B2376-50; LSBio, Seattle, MA, USA), and HSP70 monoclonal antibody (BB70; Enzo Life Sciences, Farmingdale, NY, USA) were used as primary antibodies. Samples were then incubated with a species-appropriate Alexa 488-, Rhodamine Red X-, or Alexa 647-conjugated secondary antibody (1:300; Jackson Immunoresearch, West Grove, PA, USA), after which nuclei were labeled with 4’,6-diamidino-2-phenylindol (DAPI; Invitrogen, Carlsbad, CA, USA). Sections were rinsed, mounted on slides coated with Prolong Gold Antifade reagent (Invitrogen), and examined under a Carl ZEISS-LSM 900 confocal laser-scanning microscope using ZEN software (Carl Zeiss).

**Statistics**

All experiments were performed in a blinded manner in at least duplicate. Differences in Figure 1Q, R and T were considered significant at p-values < 0.05, calculated using unpaired, two-tailed Student’s t tests using GraphPad Prism 10.0.1 (GraphPad Software Inc., La Jolla, CA, USA). The values in Figure 1Q, R and T are expressed means ± standard error of the mean (s.e.m.).

**References**

1. Xu J, Farsad HL, Hou Y, Barclay K, Lopez BA, Yamada S, et al. Human striatal glia differentially contribute to AD- and PD-specific neurodegeneration. Nat Aging. 2023;3(3):346-65.

2. Satija R, Farrell JA, Gennert D, Schier AF, Regev A. Spatial reconstruction of single-cell gene expression data. Nat Biotechnol. 2015;33(5):495-502.

3. Franzen O, Gan LM, Bjorkegren JLM. PanglaoDB: a web server for exploration of mouse and human single-cell RNA sequencing data. Database (Oxford). 2019;2019.

4. Bindea G, Mlecnik B, Hackl H, Charoentong P, Tosolini M, Kirilovsky A, et al. ClueGO: a Cytoscape plug-in to decipher functionally grouped gene ontology and pathway annotation networks. Bioinformatics. 2009;25(8):1091-3.
